# A Descriptive Cross-Sectional Assessment of Caregivers’ Knowledge and Practices Regarding the Prevention and Management of Diarrhea among Children under the Age of Five in Thulamela B Clinics, South Africa

**DOI:** 10.3390/ijerph18189452

**Published:** 2021-09-08

**Authors:** Azwinndini Ndou, Rachel Tsakani Lebese, Takalani Grace Tshitangano, Jessica Uchechi Damian

**Affiliations:** 1 Department of Public Health, School of Health Sciences, University of Venda, Thohoyandou 0950, South Africa; thophitha@gmail.com (A.N.); damjessicauche@gmail.com (J.U.D.); 2 The Office of the Executive Dean, School of Health Sciences, University of Venda, Thohoyandou 0950, South Africa; rachel.lebese@univen.ac.za

**Keywords:** diarrhea, oral rehydration therapy, under 5 mortality, hand washing, hygiene

## Abstract

Diarrhea is a common cause of child-related hospitalization and mortality among children under the age of five in South Africa. This study was conducted to assess the knowledge and practices of caregivers regarding prevention and management of diarrhea among children under the age of five in the Thulamela Municipality of South Africa. A quantitative approach using a descriptive cross-sectional survey was used. A questionnaire was adopted to collect data from caregivers at thirty primary health-care facilities using convenient sampling. Statistical Package for Social Sciences (SPSS) version 24.0 was used to analyze data. Most of the respondents have fair knowledge about diarrhea, oral rehydration therapy/salt sugar solution and its usage during diarrheal episodes. However, most of them (81.7%) do not use the salt sugar solution when their children have diarrhea. Almost all the respondents practice a hand washing hygiene for themselves and their children; 97.2% do not reheat cooked food before feeding their children; 95.5% do not drink untreated water as their source of drinking water is the municipal supply. The practices of these respondents do not reflect their knowledge in terms of the use of oral rehydration solution/salt and sugar solution. Further efforts should be made to educate caregivers on the mode of transmission of diarrheal pathogens.

## 1. Introduction

Globally, diarrhea diseases have notably been identified as the leading infectious cause of preventable death among children below the age of five. It has claimed about 1200 deaths per day mostly in Sub-Saharan Africa and South Asia, hence, “The Integrated Global Action Plan for the Prevention and Control of Pneumonia and Diarrhea” [1,2]. Moreover, within the last 10 years, South Africa was not listed as one of the 15 high-burden focus countries in the world in the pneumonia and diarrhea progress report 2020 [1]. Therefore, it is important that diarrhea is prevalent among children 0–5 years of age [3]. 

Diarrhea as a manifestation of gastrointestinal infection can be caused by different viral, bacterial and parasitic organisms [2]. Diarrheal infection may present in different ways depending on the causative pathogen. Diarrhea can be asymptomatic or mild accompanied by episodes of headaches, fever, acute abdominal pain, frequent nausea and vomiting, presence blood in stool or even death [2,4,5]. Diarrheal pathogens are commonly transmitted from one person to the other through the fecal-oral route, contaminated water or food, especially when there is poor environmental and personal sanitary hygiene [2,4]. However, literature on studies carried out in different countries indicated that diarrhea risk factors among children aged 0–5 years vary in several dimensions such as environmental, economic or social dimensions [6,7,8].

As pointed by Fagbamigbe et al. [7] in their study carried out using the Demographic and Health Survey data from 57 low and middle-income countries, health literacy was found to be an important determinant variable in diarrhea-specific mortality especially to caregivers as it can influence their health choices, behaviors and outcomes as well as that of their children. Health literacy is the ability of people to use (practices) their understanding of health information (knowledge/skill) to make informed decisions (attitudes) and actions (practices) to improve the health of their communities and their members [9]. Health literacy can help caregivers to prevent diarrhea and protect children’s health, as well as better manage diarrhea occurrences that happen. Thus, as reiterated by literature [10,11], a caregiver’s health literacy (KAP) impacts the outcome of a child’s health and wellbeing as the caregiver tends to choose and also practice behaviors that will improve several aspects of a child’s life including their nutrition, and also water and food hygiene. Improved health literacy and engaging in healthy behaviors are among the “leading health indicators for healthy people 2020” [9].

Several interventions have been in place to tackle diarrhea risk factors [12] including the use of rotavirus vaccine, support and promotion of exclusive breastfeeding at least in the first six months of a the child’s life, good community health literacy programs on how to manage and prevent diarrhea episodes and keep the children healthy through the use of oral rehydration solution (ORS) used to replace lost fluid as a result of dehydration, good hand washing with soap, use of safe drinking water and proper food and water storage containers [2,5,13,14]. According to the WHO/UNICEF [15], these proven interventions are yet to make progressive impacts to those at risk in the rural communities.

Studies [6,10] have established a significant relationship between caregiver knowledge of diarrhea and its management as well as preventive-care practices at home. In other words, the higher the health literacy level of caregivers in these studies, the lower the incidence of diarrhea episodes among the children. In the context of Thulamela B Municipality, Vhembe District, South Africa, knowledge (K) and practice (P) surveys aimed at measuring caregivers’ ability to understand and use health information on the management and prevention of diarrhea disease among children aged 0–5 years as well as form a baseline for future studies or proffer intervention [16] are needed.

This study was conducted to:

- Assess caregiver’s knowledge on the causes and danger signs of diarrhea,
- Assess caregiver’s preventive and management practices for diarrhea occurrences.

### Clarification of Concepts

Child: For the purpose of this study, a child is a person under the age of 5.

Diarrhea: In this study, diarrhea is referred to as passing of watery stools three or more times in 24 h. 

Knowledge: In the case of this study, knowledge includes caregiver’s abilities to identify the signs associated with diarrhea episodes, the causes of diarrhea, mothers’ understanding of oral rehydration therapy (ORT), salt and sugar solution (SSS) its usage.

Practices: In this study, practice refers to the frequency of the application of SSS and hygiene practices regarding food and drinking water given to children below age 5 who suffer from diarrhea.

Caregiver: For the purpose of this study, a caregiver is any person who brought the child to the clinic for consultation. 

## 2. Methods

### 2.1. Study Design

A quantitative descriptive cross-sectional survey design was adopted for this study.

### 2.2. Setting

This research was conducted in Sibasa local area (8 clinics, 1 mobile clinic and 1 healthcare Center—Thohoyandou Health Center) and Shayandima local area (9 clinics and 1 mobile clinic). In total, there were 30 primary health care facilities in the Thulamela B Municipality, Vhembe District in the Northern part of Limpopo Province, South Africa.

### 2.3. Population, Sample Size and Sampling Procedure

The target population for this study were all caregivers with children under the age of five who brought their children to any of the three primary health-care facilities clinics for consultation) in the Thulamela B Municipality: Shayandima and Sibasa Clinics and Thohoyandou Health Center The sample size of 398 was arrived at based on the convenience sampling procedure adopted during the period of data collection. Thus, any caregiver who met the inclusion criteria was recruited to participate in the study upon consultation at the selected clinics. Only 398 participants who gave informed consent to voluntarily participate in the study were included [16]. Participants were informed that participation in the study was voluntary and that they were free to withdraw from participating at any time. The study excluded caregivers with children above age 5 who came to consult at the aforementioned clinics.

### 2.4. Data Collection Tool

The investigator compiled a structured and close-ended questionnaire using the knowledge and practice survey guidelines as documented in a WHO report. It aimed to collect information on what the selected caregivers know about diarrhea and what they do or have done with the information acquired [16]. The study-specific questionnaire was divided into three sections—Section (A): Socio-demographic profile of care givers. Section (B): knowledge of caregivers which was measured using a “Yes” or “No” or Undecided scale. Section (C): focused on diarrhea preventive practices of caregivers using a “Yes”, “No” or “Undecided” measurement scale.

### 2.5. Validity

To ensure content validity of the instrument, a draft questionnaire was submitted to the supervisor for expert scrutiny regarding the relevance and readability of each item and to ensure that it was in line with the KAP model. The literature review assisted in the use of correct information in modifying the questionnaire to address the study’s aim and objectives. This was done to avoid mismatches between what the study sought to achieve and how that was supposed to be conducted. The data collection instrument was pre-tested to ensure that ambiguities were eliminated in such a way that respondents were able to comprehend the statements well. Respondents were given time to go through the questionnaire, and the researcher cleared any misunderstandings where questions arose.

### 2.6. Reliability

Reliability was ensured by pre-testing and re-testing of the data collection instrument by requesting neutral caregivers with the same characteristics to complete the questionnaire on two different occasions. Ten percent (10%), which is equivalent to 37 of the sampled caregivers from Sterkstroom Clinic, were requested to complete the questionnaire for pre-testing purposes. The reliability of the instrument was assessed by checking the similarity of responses from 37 caregivers who availed themselves for the second time. Thereafter, data were collected using this instrument. Further reliability was ensured through revising and adding items to the questionnaire, thus widening the scope of the study. It is worth noting that all the caregivers who completed the pre-testing question were excluded when the final questionnaire was completed. The term “Respondent” was used in place of real names to ensure confidentiality, anonymity and protection from harm.

### 2.7. Ethical Clearance

The Research and Innovation Directorate of the University of Venda issued an Ethical clearance certificate (SHS/18/PH/11/3005), and Limpopo Provincial Government Department of Health permission (LP_2018_006) as well as Vhembe District permission to access health care facilities. Information regarding the purpose, significance and the voluntary nature of participation were conveyed to the respondents and written informed consent was obtained from them before the administration of the instrument.

### 2.8. Data Collection

Data collection was completed using self-administered close-ended questionnaire papers. The questionnaire was developed in English and translated into Tshivenda by a language expert (for those caregivers that are not comfortable doing it in English) for data collection as most of Thulamela B residents are Tshivenda speakers. The questionnaire was transcribed back into English for analysis. 

### 2.9. Data Analysis

Data were coded, captured in an Excel spreadsheet and later analyzed using the Statistical Package for Social Sciences (SPSS) version 24.0 (IBM, Armonk, NY, USA). Descriptive statistical analysis was used to summarize data while frequency tables and charts were used to present results of the study. The responses to the knowledge section were computed into percentages and frequencies.

## 3. Results

A total of 398 were selected as participants, of which 398 were successfully interviewed. This showed a 100% response rate in the data collection process. The results presented are on the knowledge and practice of caregivers regarding diarrhea and its preventive practices for food and water.

### 3.1. Demographic Characteristics of Caregivers

Results show that the majority of the caregivers were female at 99% and their male counterparts at 1%. Data showed that 58.9% of the respondents had completed tertiary education and 31.9% had passed grade 12. Respondents with only primary and secondary level qualifications accounted for four percent (4%), respectively, followed by one percent (1%) of those with no formal qualification as shown in Table 1. Of the respondents, 92.2% were Tshivenda speaking as this study was conducted in Vhembe District where they are a dominant ethnic group. They were followed by the Tsonga speaking ethnic group at 3.8%, English 2.4% and Sotho at 1.6%.

### 3.2. Caregiver Knowledge of Diarrhea in Children <5 Years

Results revealed that the majority of the respondents identified fever (91.7%), abdominal pain (73.6%), cramps (83.6%), unformed stools (69.3%), presence of blood in the stool (94.2%) and nausea and vomiting (30%) as signs associated with diarrhea in children <5 years. In relation to the causes of diarrhea, the majority of the respondents (62.8%) identified worm infection, 93.2% identified that diarrhea is caused by germ infection, 76.4% knew that indigestible food can also result to diarrhea episodes, 63.1% affirmed to the fact that poor hygiene practices can cause diarrhea as well as teething which 95.5% identified as a major cause of diarrhea. About 51.8% of respondents had never heard of ORT/SSS, whereas 69.8% knew what ORT/SSS is used for. However, about 81.7% of respondents had never used ORT/SSS. Table 2 below shows the details.

### 3.3. Caregiver Practices Regarding Prevention and Management of Diarrhea for Children <5 Years

Study findings as shown in Table 3 and Table 4 revealed that 86.9% of the respondents allow their children to feed on their own while 13.1% do not do so. This might be as a result of age difference between the children. Regarding hand hygiene, 80.7% attested that they wash their child’s hands before feeding, while almost all the respondents (96.5%) confirmed that they usually wash theirs before feeding their children as opposed to 3.5% that never do. Regarding food safety, 97.2% do not heat up cooked foods before feeding their children and 2.8% always do. Of the respondents, 69.6% also buy food from street vendors for their children while 30.4% do not practice that. With regard to practices of caregivers in relation to types of drinking water used at home, the majority of respondents indicated that they do not use filtered water (96%) and boiled water (87%). Respondents, however, indicated that they do not use untreated water for drinking (99%), but any other type of water is used for drinking (80%). The result shows that the majority of the respondents source their water for household usage from taps (95.5%) and (97.0%) from other sources not listed in the questionnaire. The minority of the respondents indicated streams (2.8%), dams (3.3%), rainwater (4.0%), boreholes (4.3%) and rivers (4.8%). 

## 4. Discussions

Most of the study population knows about the causes of diarrhea. Indigestible foods (76.4%) were identified as the major cause of diarrhea followed by teething (75.4%), poor hygiene practices (63.1%) and worm infestation (62.8%). The report is consistent with studies carried out in India [17,18]. Only 6.8% of respondents cited germ infection as a causative factor for diarrhea in children 0–5 years, a result which agrees with a research in Lesotho [19] but contrary to the Chennai study [17] where higher respondent’s percentage (42.18%) of the study population agreed that germ infection was a major cause of diarrhea in children aged 0–5 years. The discrepancies in results might be as a result of the setting as reported by [19] whose report in the rural and urban settings presented contradictory results in relation to germ infection as a cause of diarrhea. In corroboration with other studies [4,17,18,20], caregivers (69.3%) cited their understanding and some of the signs associated with diarrhea to be, passing of unformed or watery stool more than three times a day, presence of blood in the stool, abdominal pain as well as nausea/vomiting.

According to the pneumonia and diarrhea progress report [1], ORT/SSS is one of the indicators for the Integrated Global Action Plan for the Prevention and Control of Pneumonia and Diarrhea. Though 69.8% of caregivers in this study have the knowledge of what ORT/SSS is used for, only 18.7% practice its usage on their children during diarrhea episodes. This same trend was found in a similar hospital cross-sectional descriptive study in Bangladesh [21] where there was little relationship between maternal knowledge about ORT/SSS and the maternal practice in the management of diarrhea at home.

The listed major sources of water for the respondents in this study includes, but is not limited to, taps (95.5%), rivers (4.8%), boreholes (4.3%), rain water (4.0%), dams (3.3%) and streams (2.8%). Caregivers were probed about their hygiene practices with respect to food and drinking water at home as a measure of diarrhea management and prevention. The majority (86.9%) cited that their children feed by themselves, and also with their hands washed before feeding (80.7%). In addition, there is good hand hygiene practice by caregivers as 96.5% cited that they usually wash their own hands before feeding their children. These findings were supported by literature, which indicated a good percentage of the study participants reportedly wash their hands before feeding their children [18,19].

In this study, caregivers did not reheat cooked or leftover food before feeding (97.2%) including those that buy food from street vendors to feed their children (30.4%) are more likely to develop diarrhea because as indicated by the WHO [2], improper food handling can result in contamination with pathogens that can cause diarrhea in children. Although 96.2% of the respondents do not drink filtered water nor boiled water (87.4%), almost all (98.7%) reported that they do not give their children untreated water. This may be due to the fact that 95.5% source their drinking water from municipal taps at home. A major public health concern with regards to caregiver practices with drinking water are the reports of positive cases of sapovirus as well as a high distribution of *Enterococcus faecalis* and *Eschericia. coli* as fecal coliform bacteria in all the selected drinking water samples (municipal taps and boreholes, rivers and springs) collected at the point-of-use in study settings located in Vhembe District across all seasons [4,22].

## 5. Conclusions

Although several studies have established the significant relationship that exists between the level of health literacy and their behavioral outcomes towards the management and prevention of diarrhea among children under the age of five, this paper does not validate such established fact. Findings in this study revealed that despite the fact that more than half (58.9%) of the caregivers who participated in this study attained tertiary education level, had a fair knowledge about diarrhea (with regard to the observable signs, the use of oral rehydration therapy/salt and sugar solution), they do not really know what causes diarrhea. Their knowledge about oral rehydration therapy/salt and sugar solution (69.8%) are not translated into practice (18.3%) since more than half (73.1%) of the caregivers noted that their children had diarrhea 14 days prior to the data collection day; in other words, they must have used ORT/SSS so as to prevent dehydration. These findings may be interpreted to mean that having knowledge about a health condition and remedy alone is not enough to prompt expected action [23]. There is need for proper health literacy on diarrhea, its causes, management and prevention.

### 5.1. Recommendations

Health literacy should focus on the causes of diarrhea, preparation and benefits of ORT/SSS. In addition, in-depth study into municipal water as the main cause of diarrhea in that Vhembe District should be considered since previous studies reported positive cases of diarrhea pathogen in water samples collected at the point of usage in homes. Further studies should be done to ascertain the relationship between socio-demographic characteristics of caregivers in this setting with their knowledge and practice with regards to diarrhea management and prevention among children between the ages of 0 and 5 years. Other studies should be carried out on the alternative remedies adopted by mothers in this setting for diarrhea management among children below age five.

### 5.2. Strength and Limitations

The strength is that there was no similar documented study in this setting. Thus, this study can be used as a baseline for other studies as well as a blueprint for interventional studies in Thulamela B Municipality. The limitation is that the study did not establish alternative home-based management methods adopted by caregivers when their children experience diarrhea, especially those that do not make use of oral rehydration solutions. The study did not establish the relationship between respondent’s socio-demographic characteristics and its relationship with their knowledge and practice of diarrhea management and prevention among children between the ages of 0 and 5 years. The study did not translate the questionnaire into Tshivenda during the pre-test and re-test; it did not make provisions for questionnaire translation to Tsonga and Sotho in order to accommodate those that are neither fluent in written English or Tshivenda.

## Figures and Tables

**Table 1:** Socio-demographic characteristics of respondents.

		Frequency	Percentage (%)
Gender	Female	394	99.0
	Male	4	1.0
Age	15–25	124	31.2
	26–35	190	47.7
	36–45	67	16.8
	46 and Above	17	4.3
Highest Qualification	No formal education	5	1.3
	Primary level	17	4.3
	Secondary level	17	4.3
	Passed grade12	127	31.9
	Tertiary level	232	58.9
Home Language	English	10	2.4
	Tshivenda	367	92.2
	Tsonga	15	3.8
	Sotho	6	1.6
Marital Status	Single	203	51.0
	Married	181	45.4
	Divorced/ Separated	9	2.3
	Widow	5	1.3
Occupation	Employed	81	20.6
	Unemployed	289	72.4
	Self-employed	26	6.5
	Pensioner	2	0.5
Relationship to child	Grandmother	29	7.3
	Mother	342	85.9
	Aunt	8	2.0
	Other	19	4.8

**Table 2:** Caregiver knowledge regarding causes and danger signs of diarrhea in Children <5 Years.

		Frequency	Percentage (%)
Do you know what diarrhea is?	No	205	51.5
	Yes	193	48.5
Has this child suffered from diarrhea in the past 2 weeks (past 14 days)?	No	107	26.9
	Yes	291	73.1
**Knowledge about causes of diarrhea in children 0–5 years**			
Worm infection	No	148	37.2
	Yes	250	62.8
Germ infection	No	371	93.2
	Yes	27	6.8
Indigestible foods	No	94	23.6
	Yes	304	76.4
Poor hygiene practices	No	147	36.9
	Yes	251	63.1
Teething	No	98	24.6
	Yes	300	75.4
**Knowledge about diarrhea and some of the danger signs**			
Did your child experience nausea and vomiting during diarrhea?	No	278	69.8
	Yes	120	30.2
Did your child experience fever during diarrhea	No	33	8.3
	Yes	365	91.7
Did your child experience abdominal pain?	No	105	26.4
	Yes	293	73.6
Did your child experience cramps?	No	67	16.8
	Yes	331	83.2
Did your child pass three or more unformed stools during the day?	No	122	30.7
	Yes	276	69.3
Did your child have blood in the stool?	No	23	5.8
	Yes	375	94.2
Did your child show any other signs that are not listed here?	No	299	75.1
	Yes	99	24.9
Do you think diarrhea is dangerous?	No	18	4.5
	Yes	380	95.5
**Knowledge about ORT/SSS**			
Have you heard of ORT/SSS?	No	206	51.8
	Yes	192	48.2
Do you know what ORT/SSS is used for?	No	120	30.2
	Yes	278	69.8
Have you ever used ORT/SSS before?	No	325	81.7
	Yes	73	18.3

**Table 3:** Maternal practices for food and water safety in the prevention of diarrhea for Children <5 Years.

Practices of Mothers Regarding Feeding and Hygiene Aspects.		Frequency	Percentage (%)
Does your child usually feed on his or her own?	No	52	13.1
	Yes	346	86.9
Do you wash your child’s hands before he/she eats?	No	77	19.3
	Yes	321	80.7
Do you wash your hands before feeding your child?	Never	14	3.5
	Usually	384	96.5
Do you warm cooked food before you feed your child?	Always	11	2.8
	No	387	97.2
Do you buy food from street vendors for your child?	Yes	121	30.4
	No	277	69.6
**Practices of mothers regarding types of drinking water used at home.**			
Filtered water for drinking.	No	383	96.2
	Yes	15	3.8
Untreated water for drinking.	No	393	98.7
	Yes	5	1.3
Boiled water for drinking.	No	345	87.4
	Yes	50	12.6
Other types of water for drinking.	No	76	19.6
	Yes	320	80.4

**Table 4:** Respondents’ sources of drinking water.

Source of Water	Frequency	Percentage (%)
River	19	4.8
Tap	381	95.5
Stream	11	2.8
Rain water	16	4.0
Borehole	17	4.3
Dam	13	3.3
Other sources	386	97.0

## Data Availability

The study did not report any archived dataset.
